# Inaccessible rocky cliffs: An optimized method for plant data collection in extreme environments

**DOI:** 10.1016/j.mex.2019.05.021

**Published:** 2019-05-22

**Authors:** Estrella Alfaro-Saiz, Víctor Granda, Alberto Rodríguez, Raquel Alonso-Redondo, Marta Eva García-González

**Affiliations:** a Department of Biodiversity and Environmental Management, University of León, Spain; b CTFC Forest Science Centre of Catalonia, Spain

**Keywords:** Grid-cells data collection for plants, Minimal effort, Data collection optimization, Rupicolous flora, Highest accuracy, Inaccessibility

## Abstract

Counts are normally used to assess the densities of plants. However, due to the physical characteristics of these sites, habitats and species associated with inaccessible rocky cliffs and other extreme environments pose additional challenges. It is therefore necessary to apply changes to the usual data collection methods. This system allows population sizes to be estimated from an incomplete data collection. This is important because when data collection sites are inaccessible, the fieldwork cannot be carried out within the time that is normally allocated. Furthermore, the minimum sampling effort involved enables economic resources to be saved. This method allows the time spent and the material, methodological and human resources used to be reduced while simultaneously allowing the highest level of accuracy to be maintained.

• The minimum effort needed to carry out data collection of plants on vertical walls and other difficult-to-access environments is calculated.
• The proposed method is based on the search for the theoretical distribution function with a better adjustment to the actual distribution of the studied species.
• This system allows to reduce the necessary resources, while the maximum accuracy is maintained in the calculations.

## Graphical abstract

**Figure:**
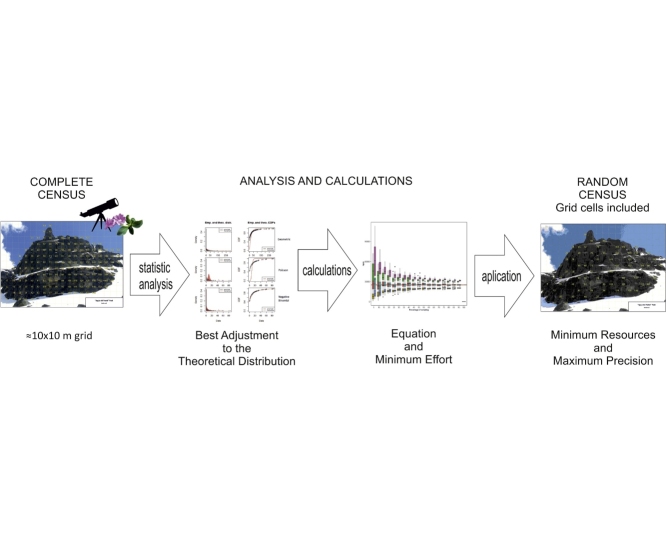

**Specifications Table**

**Table:** 

**Subject Area:**	*Environmental Science*
**More specific subject area:**	*Botany*
**Method name:**	*Grid-cells data collection for plants*
**Name and reference of original method:**	**García *et al.* 2002; García-Baquero *et al* 2002; Goñi *et al.* 2006;** García MB, Guzmán D, Goñi D (2002) An evaluation of the status of five threatened plant species in the Pyrenees. Biol Conserv 103:151-161. García-Baquero G, Herrera M, Amat De León E, Beamonte E, Dato M, González R, Gordiola F, Martínez-Zaporta JL, Muñoz I, Ruiz De Alda (2002) Estimación de la abundancia de *Androsace rioxana* A. Segura. Stvdia Botanica 21: 139-142. Goñi D, García MB, Guzmán D (2006) Métodos para el censo y seguimiento de plantas rupícolas amenazadas. Pirineos 161:33-58.
**Resource availability:**	*NA*

## Method details

### Data collection

**Fig. 1:**
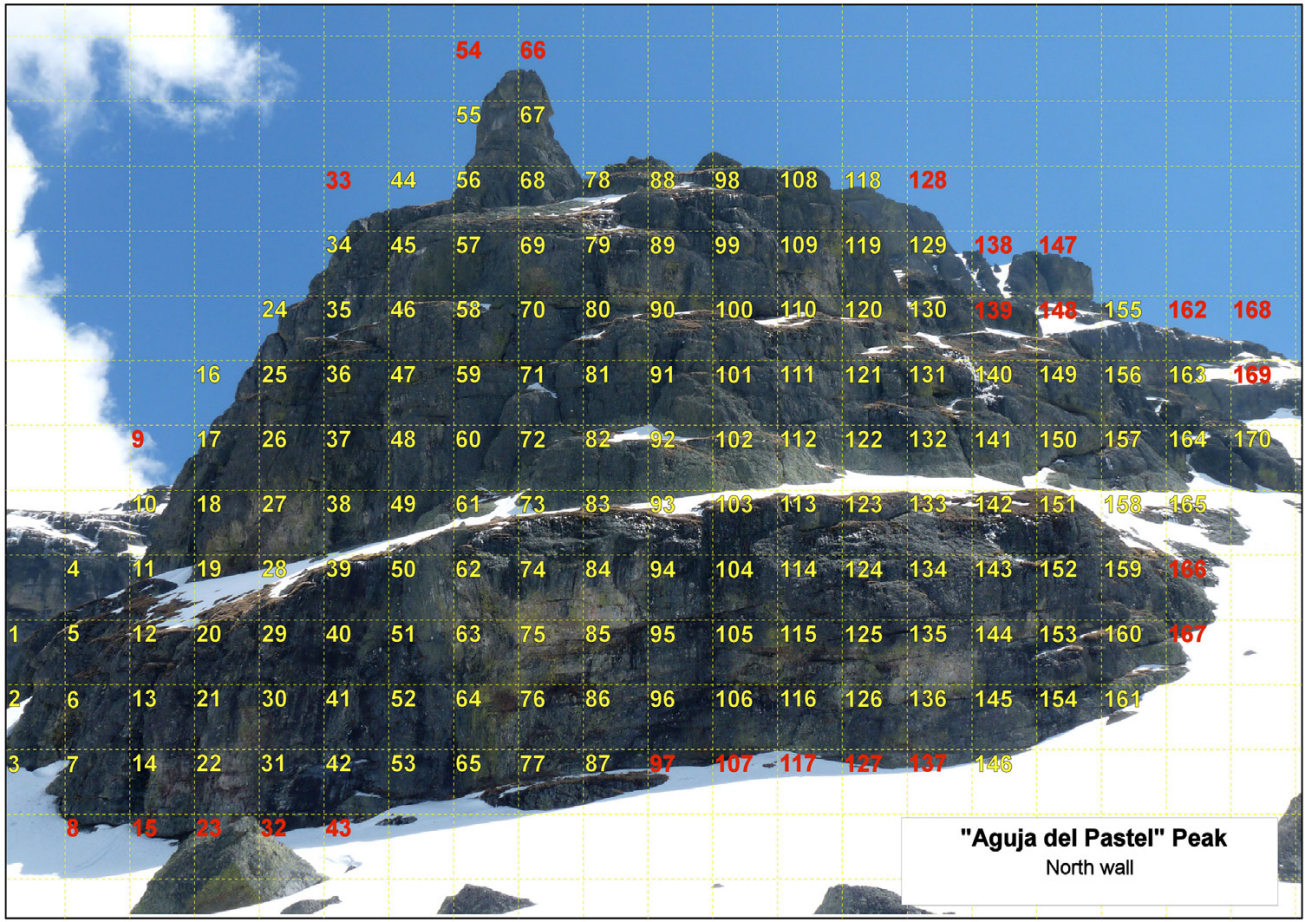
Image of a cliff. It has been treated through the use of Geographic Information System (GIS) software. The dimensions of the image correspond to the real distance. A 10 × 10 m grid was then added to the image, assigning an individual identification code to each of the grid-cells. Red numbers represent the cells that were removed to avoid the edge effect.

The first step is to locate the populations or population nuclei in which the data collection will be carried out. Once the target cliffs to be monitored have been selected, a photograph is taken of each one, seeking the maximum perpendicularity of the wall. To minimize the spatial deformation, if the cliff is very wide, several photos are taken at regular distances. The image is measured using Geographic Information System (GIS) software and the picture is adjusted so the dimensions correspond to the actual distance, following the method developed by Goñi et al. [1]. To explain this method we have selected as an example the north wall of “Aguja del Pastel” Peak. This vertical wall is located in the Curavacas Massif (Palencia, Spain). The area of the studied wall is 1470 m^2^ approximately. A 10 × 10 m grid is then added to the image, assigning an individual identification code to each of the grid-cells (Fig. 1). In the case study, the grid-cells at the extremities, which showed a very small percentage of rock in the photograph, were eliminated to avoid the edge effect.

To carry out data collection on rocky cliff faces, the authors propose an adaptation of the methodology for rupicolous species based on counting units using optical devices [2,1]. The counting units are termed “visual units”. These correspond to the number of individuals that can be observed with the naked eye using the optical devices employed for this purpose. A 20×60× terrestrial telescope was used to conduct the data collection. Depending on the final objective of the data collection, the choice of these “visual units” may differ. This method can be applied to the whole population, or it can be applied only to the data collection of mature individuals [3]. To counteract errors linked to the observer and to adjust for bias, as well as to avoid underestimating or overestimating the population, a correction factor – CF – was used [2,1]. The CF measures the relationship between the number of “visual units” observed using optical devices and the actual count in accessible areas. The inability to find enough accessible areas on rocky cliff faces, a necessary requirement to calculate the CF, led the present authors to adapt the calculation to inaccessible rocky cliff face conditions. To do so, between 30 and 40 easily delimited areas had to be selected where the observers carried out two counts: one with the same lens used for the original data collection (20×) and the other with a higher magnification lens, which enabled individuals to be counted with greater precision (60×). If there were enough accessible areas, the ratio between the real number of individuals and the number of “visual units” could be used to estimate the total number of individuals from the number of visual units recorded in inaccessible areas or populations [2]. This procedure must be repeated for each observer and for the different sampling years. In this case, the distance from the observers to the cliff was 400 m. The ratio between both measurements (20×/60× or real number of individuals/number of visual units) is used to estimate the CF. The data obtained must be adjusted to a normal distribution in order to calculate the mean. The average was multiplied by the total of “visual units” recorded for each grid-cell.

For subsequent calculations, a data collection of at least the full cliff face is recommended.

### Adjustment of the theoretical distribution model

Once the complete results of the data collection had been obtained by adding the individual results of each of the grid-cells, the data were analyzed using the MASS package [4] and the fitdistrplus package [5] from R statistical software [6]. Other R packages were used to perform the necessary operations (see Supplementary material -S1-): readxl [7], dplyr [8], tidyr [9] and ggplot2 [10].

Firstly, the best adjustment of the data to possible distributions was determined. Different theoretical distributions that could explain the real distribution of the species were compared [11]. In the present case the data were adjusted to the following theoretical distributions: Geometric, Poisson and Negative Binomial. The data were adjusted to the different distributions in each case. To assess which theoretical model the data were best suited to, the observed frequencies and the theoretical frequencies were compared. To this end, a graphical analysis and goodness of fit Chi-square (χ²) test were performed. The following initial hypothesis (H_0_) is proposed: the observed frequencies did not significantly differ from the expected frequencies. H_0_ is rejected if the p-value obtained was less than the chosen significance level for the test (0.05).

In order to make a numerical evaluation of the model that exhibited the best adjustment, the indices Aikake's Information Criterion – AIC – [12] and Bayesian Information Criterion – BIC – [13] had to be analyzed. Models that exhibit a lower value of these indices are regarded as a better fit.

**Fig. 2:**
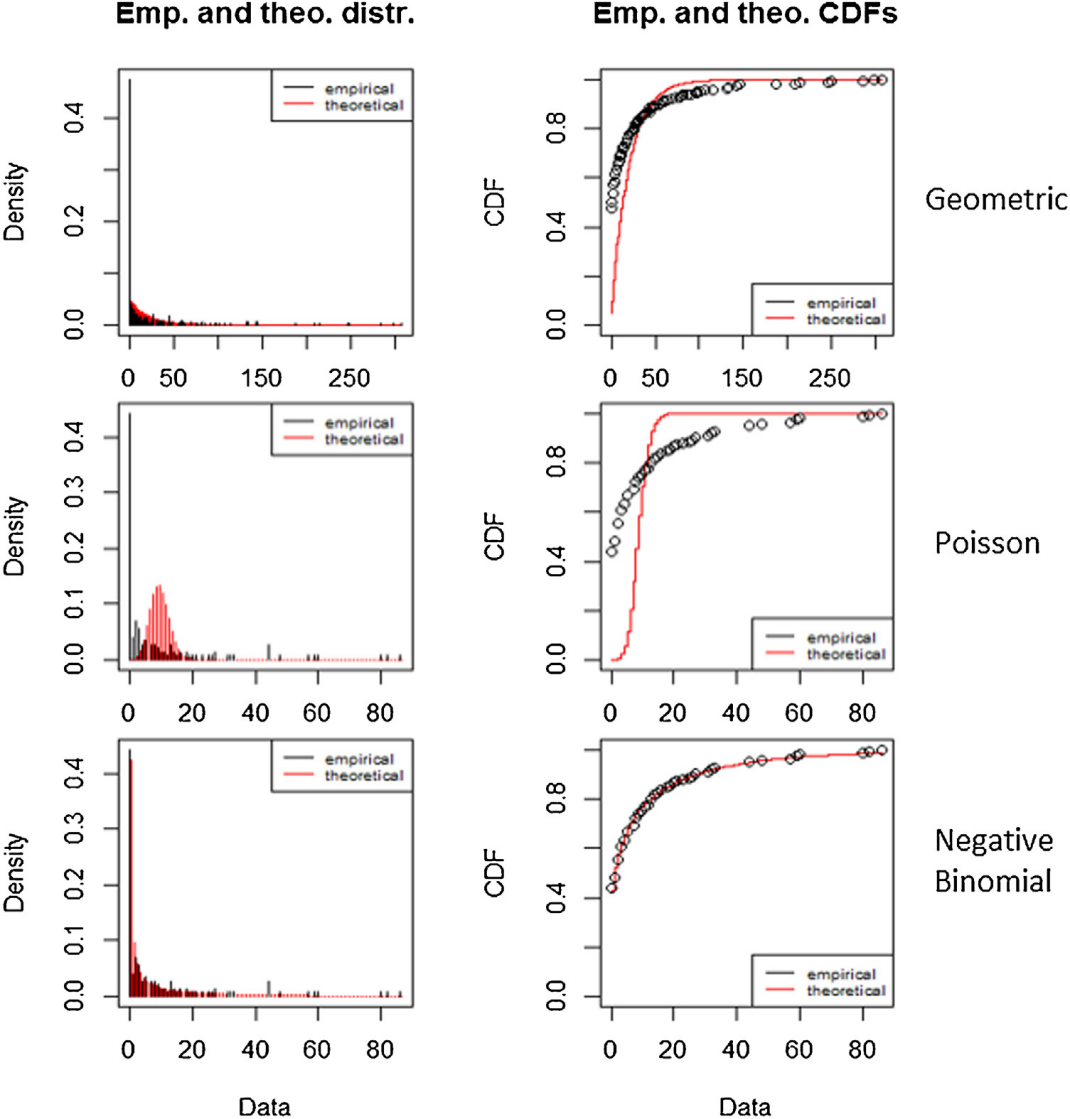
Probability density adjusted to a Geometric, Poisson and Negative Binomial theoretical functions and CDF (Cumulative Distribution Function) empirical and theoretical. Red lines represent theoretical data and black lines represent empirical data.

**Fig. 3:**
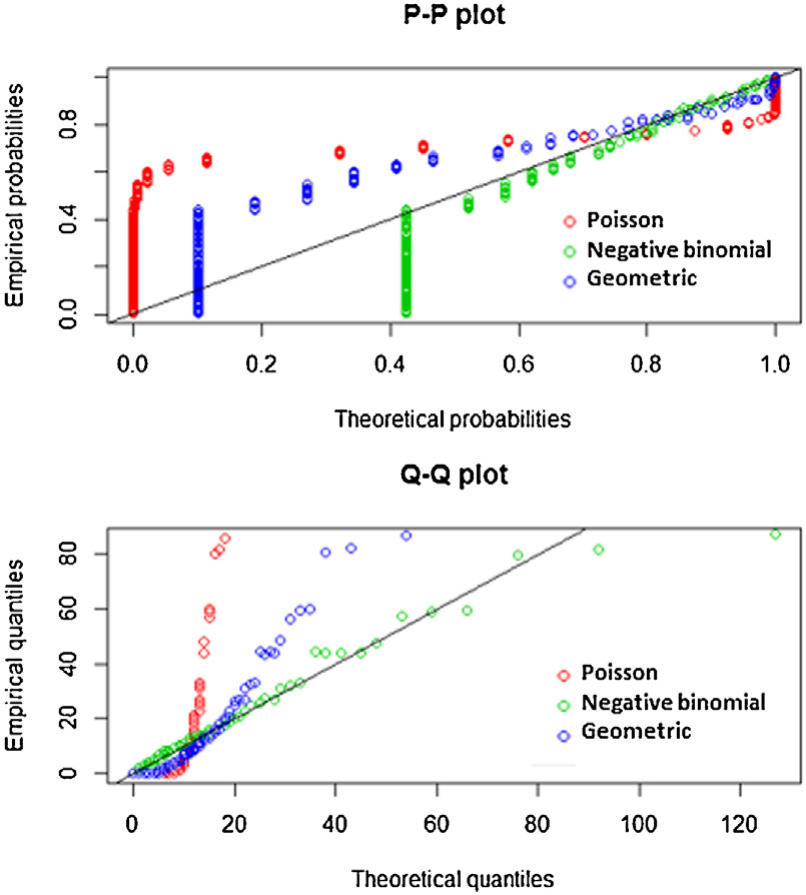
PP and QQ empirical and theoretical plots.

The graphical evaluation was carried out by analyzing the Cumulative Distribution Function – CDF – (Fig. 2), PP Plot and QQ Plot graphics (Fig. 3). PP Plot graphics were built from the empirical distribution function of the sample (x) and designed so as to represent each empirical observation versus the expected value, thereby obtaining a straight line. QQ Plot graphics represent empirical quantiles obtained in the sample versus the corresponding quantile of the distribution.

### Minimum effort calculation

Taking into account that the final result (the result of the completed data collection) was known, the authors proceeded to determine the smallest sample size that could be stipulated in order to obtain a reliable measure of population size, thus determining the minimum effort required to carry out the data collection. In order to do so, the total data were adjusted to the distribution that presented the best adjustment and the parameters of this distribution were thus obtained. Subsequently, different data subsets were created, gradually reducing the sample sizes and then calculating the estimated population number to compare it with the real number and also the confidence intervals (CI). The percentage of plots sampled was reduced by 5% each time, starting at 90% and ending at 5%, always selecting plots at random.

CI values were calculated using two methods: “Maximum Likelihood” (ML) and “Simple Bootstrap” (SB). ML is an approximation to the normal logarithm transformation of the variable. This method makes it possible to find the probability distribution that makes the observed data more likely. SB is a method for calculating CI in which the initial sample (the percentage of grid-cells sampled) was resampled with replacement [[14], [15], [16]]. In this case, the initial sample was resampled 10,000 times. The process was repeated 25 times for each of the plots sampled.

**Fig. 4:**
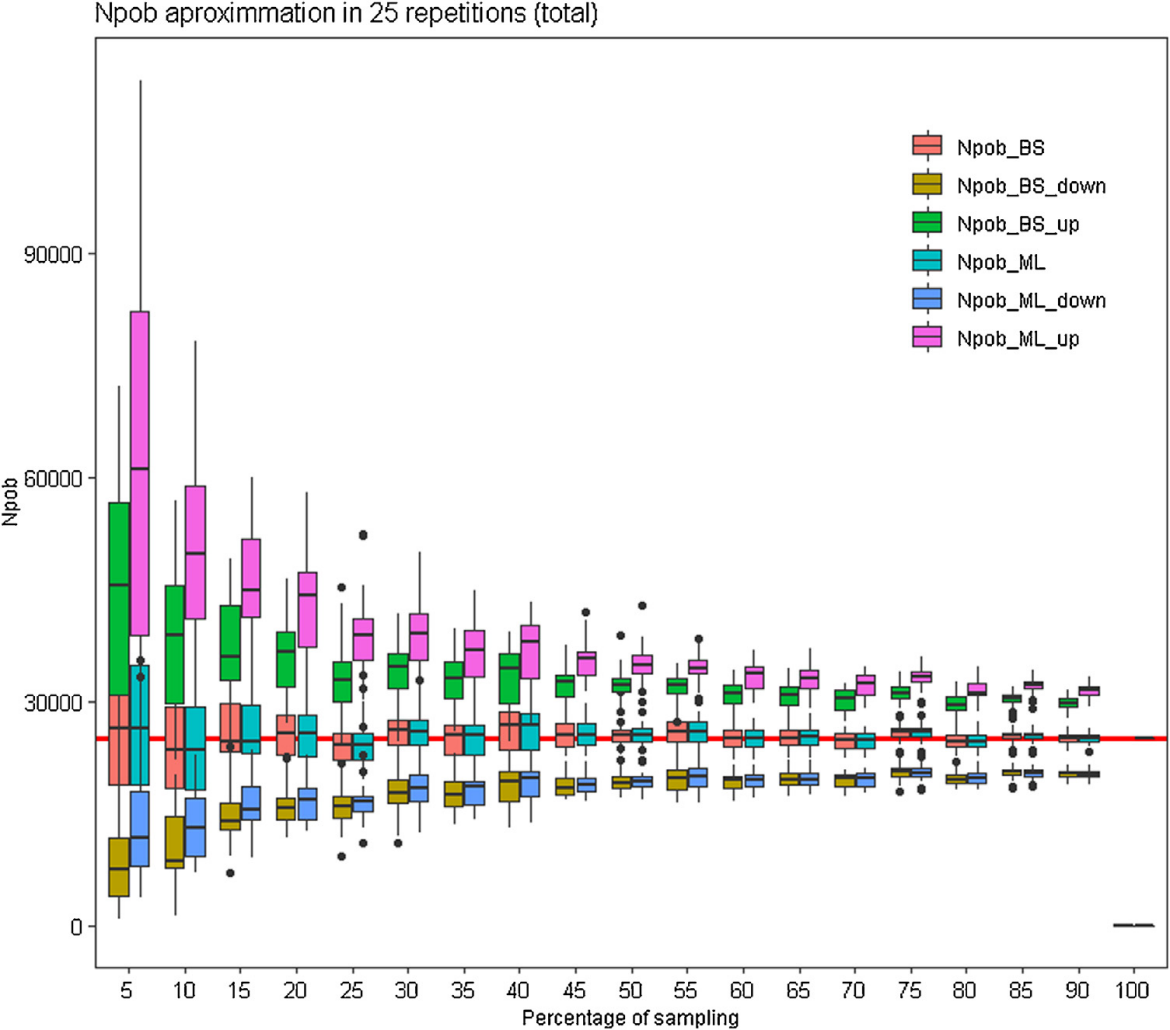
Confidence Intervals (CI) calculated for each percentage of random sampling using two methods: “Simple Bootstrap” (BS) and “Maximum Likelihood” (ML). The red line represents the total population size.

**Fig. 5:**
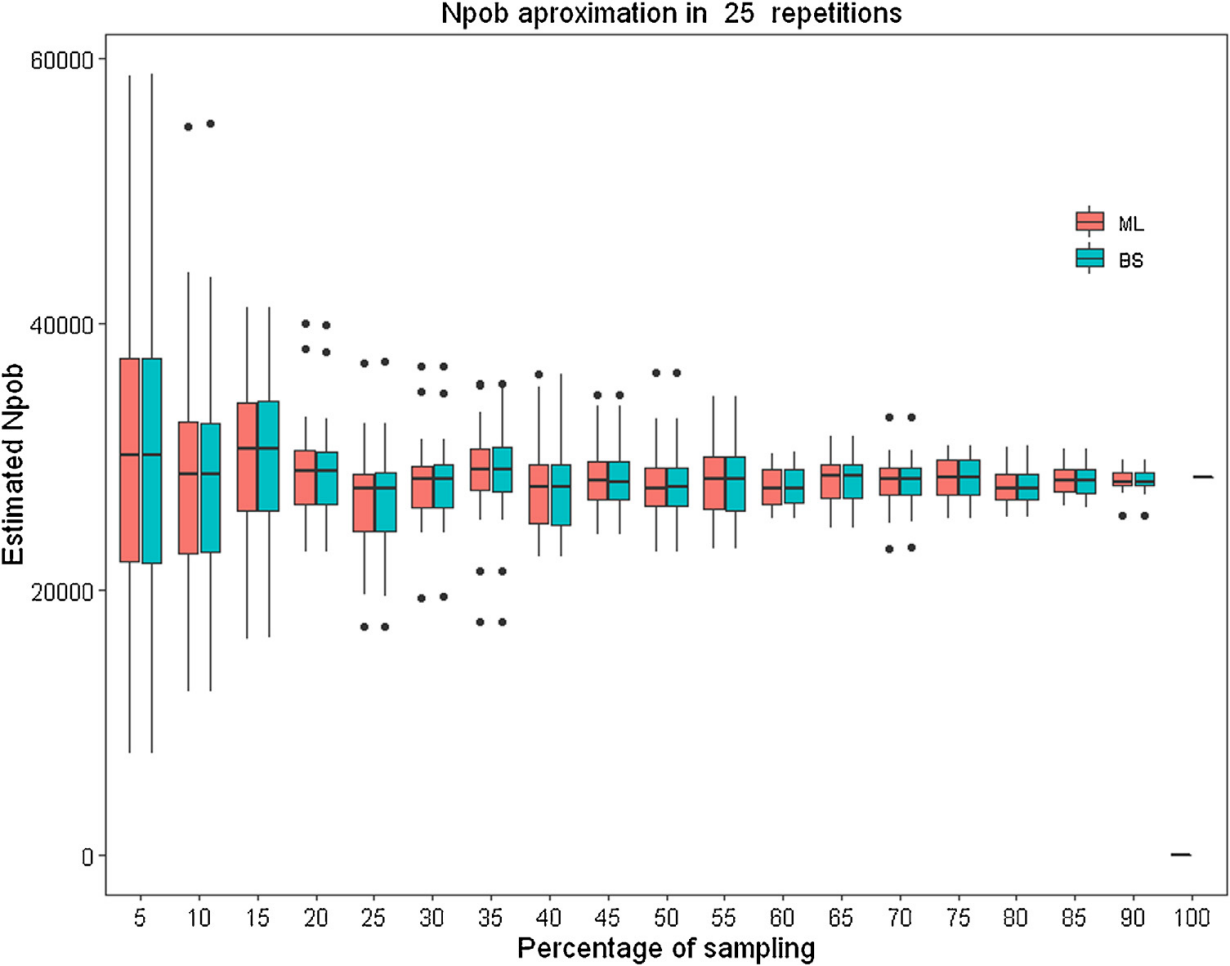
Range of mean values obtained with “Simple Bootstrap” (BS) and “Maximum Likelihood” (ML).

**Fig. 6:**
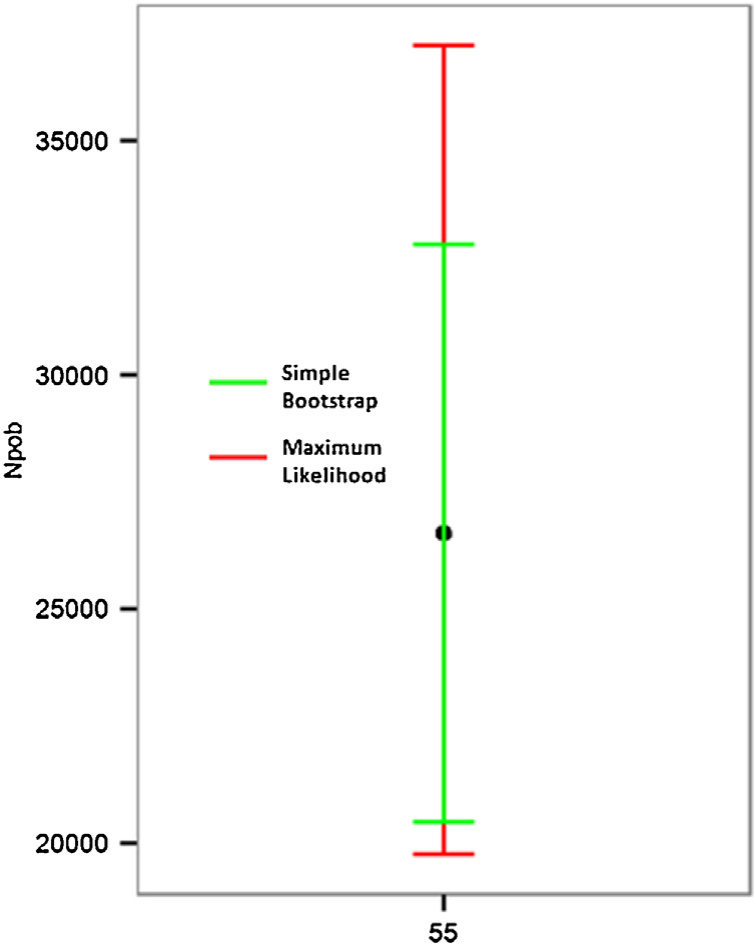
The CI values obtained for a previously selected sampling value (55%) with “Simple Bootstrap” (BS) and “Maximum Likelihood” (ML).

The results are represented by a series of boxplots (box and whisker plot) in which CI values (mean, maximum and minimum) appear as well as the estimated population number calculated for 25 repetitions (Fig. 4). For each dataset, the most exact CI calculation method would be selected, that is to say, the one with the smallest error bar. In Fig. 4, the boxplot diagrams make it possible to visually determine that smaller intervals were obtained using the two methods and how CI are reduced as the sample rate is increased. Fig. 5 represents the range of mean values obtained with the two proposed methods. Fig. 6 represents the CI obtained for a previously selected sampling value (55% in these examples). After performing a detailed analysis, the method with the lowest CI must be selected.

A graphical analysis allows the determination of the minimum sampling effort that can be applied to carry out a data collection of each specific real case while maintaining the highest possible accuracy in the calculations. This method also enables the population number to be calculated, based on incomplete information. This is very useful in sampling areas where the weather is very variable. It would allow the population to be inferred if the data collection could not be completed. The proposed method could be very useful in other experimental methodologies that involve inaccessibility and/or extreme environments. This method was previously applied in the article published by Alfaro-Saiz et al. [17].

## Appendix A Supplementary data

Appendix S1 contains the R code to facilitate the application of this method by other scientists.
