# Supplementary material for: Inaccessible rocky cliffs: An optimized method for plant data collection in extreme environments
Source: MethodsX. 2019 May 22;6:1199–206. doi: 10.1016/j.mex.2019.05.021 (PMC6541732; doi:10.1016/j.mex.2019.05.021)
Supplement: Supplementary file 1 [file mmc1.pdf]

Supplementary material
R Scripts

```
####Packages####

library(MASS)
library(readxl)
library(dplyr)
library(tidyr)
library(fitdistrplus)
library(ggplot2)


#### Data ####

data_cf <- read_excel('CF.xlsx', 1)
data_census<- read_excel('datatable.xlsx', 1)
#CF.xlxs and database.xlsx are table names#


#### Mean ####

media_cf<- data_cf %>%
  mutate(cf = cerca_60x/lejos_20x) %>%
  summarise_all(funs(mean))


#### Normalization of the census data with the correction factor ###

data_census_normalized_cf <- data_census%>%
  mutate(N_normalized = N_individuals*0.884)%>%
  slice(-230)

#### Simulation ####

# datos para ajustes#
# total (este+norte)#
total_population<- round(as.vector(data_census_normalized_cf $N_normalized), 0)


# Adjustment of the data to different theoretical distributions #

total_population.pois.fit <- fitdist(total_population, 'pois', method = 'mle')
total_population.nbin.fit <- fitdist(total_population, 'nbinom', method = 'mle')
total_population.geom.fit <- fitdist(total_population, 'geom', method = 'mle')


# Summary and plots #

summary(total_population.pois.fit)
summary(total_population.nbin.fit)
summary(total_population.geom.fit)

plot(total_population.pois.fit)
plot(total_population.nbin.fit)
plot(total_population.geom.fit)

cdfcomp(list(total_population.pois.fit,total_population.nbin.fit,total_population.geom.fit))
denscomp(list(total_population.pois.fit,total_population.nbin.fit,total_population.geom.fit))
qqcomp(list(total_population.pois.fit,total_population.nbin.fit,total_population.geom.fit))
ppcomp(list(total_population.pois.fit,total_population.nbin.fit,total_population.geom.fit))
```

```r
#Goodness of fit of the theoretical models #

gofstat(list(total_population.pois.fit,total_population.nbin.fit,total_population.geom.fit))


#### COMPLETE ANALYSIS WITH SIMULATION ####

#Source ('Scripts/Simulation_script.R')#

source('Scripts/Better_simulation_script.R')

sim_total_population <- RepCISimulation(total_population, rep = 25, bootstrapN = 10000)
sim_total_population
sim_total_population.df <- sim_total_population$tabla

sim_total_population$PlotPob
sim_total_population$PlotCIUp
sim_total_population$PlotCIDown


# Plots #

sim_total_population_Npob <- sim_total_population.df %>%
  dplyr::select(Percentage, Npob_ML, Npob_BS) %>%
  tidyr::gather(method, Npob, Npob_ML:Npob_BS)
sim_total_population_Npob$Percentage <- as.factor(sim_total_population_Npob$Percentage)
sim_total_population_up <- sim_total_population.df %>%

  dplyr::select(Percentage, Npob_ML_up, Npob_BS_up) %>%
  tidyr::gather(method, Npob_up, Npob_ML_up:Npob_BS_up)
sim_total_population_up$Percentage <- as.factor(sim_total_population_up$Percentage)

sim_total_population_down <- sim_total_population.df %>%
  dplyr::select(Percentage, Npob_ML_down, Npob_BS_down) %>%
  tidyr::gather(method, Npob_down, Npob_ML_down:Npob_BS_down)
sim_total_population_down$Percentage <- as.factor(sim_total_population_down$Percentage)

Npob_real_total_population <- sim_total_population_Npob$Npob[[1]]

plot_simulacion_total_population <- ggplot(data = sim_total_population_Npob,
                                           aes(x = Percentage, y = Npob, fill = method)) +
  geom_hline(aes(yintercept = Npob_real_total_population),
             size = 1.25,
             colour = "red") +
  geom_boxplot() +
  geom_boxplot(data = sim_total_population_up,
               mapping = aes(y = Npob_up)) +
  geom_boxplot(data = sim_total_population_down,
               mapping = aes(y = Npob_down)) +
  labs(title = 'Npob aproximmation in 25 repetitions (total)',
       x = 'Percentage of sampling', y = 'Npob') +
  theme_bw() +
  theme(title = element_text(size = rel(0.9)),
        axis.title = element_text(size = rel(.9)),
        axis.text = element_text(size = rel(.9)),
        panel.grid.major = element_blank(),
        panel.grid.minor = element_blank(),
        legend.background = element_blank())

plot (plot_simulacion_total_population)
```
